# Appraising growth differentiation factor 15 as a promising biomarker in digestive system tumors: a meta-analysis

**DOI:** 10.1186/s12885-019-5385-y

**Published:** 2019-02-26

**Authors:** Yanqiu Wang, Tao Jiang, Mingyan Jiang, Shuijing Gu

**Affiliations:** 1 Department of Clinical Laboratory, The First Affiliated Hospital, and College of Clinical Medicine of Henan University of Science and Technology, No.24 Jinghua Road, Jianxi District, Luoyang, 471000 China; 2 Department of Rehabilitation, Seafarers’ General Hospital in Heilongjiang Province, Harbin, 150000 China; 3 Department of Clinical Laboratory, The Fifth People’s Hospital of Haimen City in Jiangsu Province, Haimen, 226131 China

**Keywords:** Cytokine growth differentiation factor 15, Digestive system tumor, Diagnosis, Prognosis, Meta-analysis

## Abstract

**Background:**

Previous studies have highlighted cytokine growth differentiation factor 15 (GDF-15) as a potential biomarker for digestive system tumors (DST). This study sought to assess the feasibility of using GDF-15 as a diagnostic and prognostic biomarker in DST.

**Methods:**

Eligible studies from multiple online databases were reviewed. Meta-analyses of diagnostic parameters were carried out using standard statistical methods. Study-specific hazard ratios (HRs) with 95% confidence intervals (CIs) were calculated to estimate the strength of the relationship between GDF-15 levels and clinical prognosis.

**Results:**

We identified 17 eligible studies comprising 3966 patients with DST. The sensitivity, specificity, and area under the curve (AUC) for the discriminative performance of GDF-15 as a diagnostic biomarker were 0.74 (95% CI: 0.68–0.80), 0.83 (95% CI: 0.75–0.89), and 0.84, respectively. Moreover, increased GDF-15 expression levels were markedly associated with unfavorable overall survival (OS) in patients with DST (HR = 2.34, 95% CI: 2.03–2.70, *P* < 0.001; I^2^ = 0.0%) and colorectal cancer (CRC) (HR = 2.27, 95% CI: 1.96–2.63, *P* < 0.001; I^2^ = 0.0%). Stratification by cancer type, test matrix, ethnicity, and cut-off setting also illustrated the robustness of the diagnostic value of GDF-15 in DST.

**Conclusion:**

Collectively, our data suggest that GDF-15 expression level may have value as a diagnostic and prognostic biomarker, independent of other, traditional biomarkers.

**Electronic supplementary material:**

The online version of this article (10.1186/s12885-019-5385-y) contains supplementary material, which is available to authorized users.

## Background

Over the past decade, digestive system tumors (DST) have become major causes of cancer-related mortality worldwide [1]. According to global cancer statistics compiled in 2016, death rates have increased for patients with DST, including for those with liver cancer and pancreatic cancer [2]. Due to lack of sensitive diagnostic testing, large numbers of patients with DST are mostly diagnosed at advanced stages, resulting in poor 5-year survival rates [2]. It is therefore necessary to identify novel, reliable biomarkers which can predict early diagnosis and/or prognosis of patients with DST.

Human growth differentiation factor 15 (GDF-15), also known as macrophage inhibitory cytokine-1 (MIC-1), is a divergent member of the transforming growth factor-β (TGF-β1) superfamily of proteins [3, 4]. The human GDF-15 gene maps to chromosome 19 in the p13.1–13.2 region, and encodes a 25-kDa secreted growth factor that is highly expressed in cardiomyocytes, adipocytes, endothelial cells, and macrophages in both normal and diseased tissues [3–5]. Intriguingly, GDF-15 levels are substantially increased in various pathological conditions, including inflammation and injury [5–7]. Notably, experimental and epidemiological evidence has demonstrated that GDF-15 levels are up-regulated in many types of DST, such as colorectal cancer (CRC) [8–13], gastrointestinal cancer (GC) [14, 15], pancreatic cancer (PC) [16–20], esophageal carcinoma (EC) [21, 22], and liver cancer [23, 24]. Recently, GDF-15 has received much attention as a diagnostic and prognostic biomarker in DST. However, data are inconsistent among studies assessing the clinical relevance of GDF-15, and the statistical power of these studies has also been insufficient. In this study, we collected published studies regarding the expression of GDF-15 in DST and performed a meta-analysis to determine whether high GDF-15 expression levels can be used as a diagnostic or prognostic biomarker in DST.

## Methods

### Literature search

We searched the PubMed, EMBASE, ESBCO, Wiley Online Library, and Ovid databases for eligible studies from their incipience to June 20, 2018. We used the following search terms or Medical Subject Headings (MeSH) words to identify eligible studies: “macrophage inhibitory cytokine-1/MIC-1/growth differentiation factor 15/GDF-15” AND “oesophageal cancer/oesophageal neoplasm/colorectal cancer/colorectal carcinoma/colon cancer/colon carcinoma/CRC /gastrointestinal cancer/gastric carcinoma/gastric cancer/stomach cancer/hepatocellular carcinoma/liver cancer/pancreatic carcinoma/ pancreatic neoplasms/pancreatic ductal adenocarcinoma/pancreatic mass/digestive system tumor/digestive system neoplasm” AND “survival/prognosis/outcome/hazard ratio/HR” OR “diagnosis/sensitivity/specificity/ROC/AUC/area under the curve”. Reference lists of the included articles or relevant reviews were also browsed for potentially missing studies.

### Inclusion and exclusion criteria

Studies meeting the following criteria were included: (1) clinical trials reporting the diagnostic and/or prognostic features of GDF-15 in DST; (2) studies where the diagnostic parameters or survival outcomes included sensitivity, specificity, area under the curve (AUC), overall survival (OS), disease free survival (DFS), progression-free survival (PFS), recurrence-free survival (RFS), tumor-specific survival (TSS), or cancer-specific survival (CSS); and (3) the estimated hazard ratios (HR) or odds ratio (OR) with corresponding 95% confidence intervals (CIs) were available or could be calculated from published data. Accordingly, exclusion criteria included: (1) studies defined as reviews, basic studies, animal studies, letters, or conference abstracts; (2) data for statistical analyses were unavailable, and also failed to contact the authors; (3) studies with high risk and bias in quality assessment; and (4) articles written in a language other than English.

### Data extraction and quality assessment

Data extraction was performed for study sensitivity, specificity, sample numbers, as well as HRs and their corresponding 95% CIs. Where such data were unavailable, the values were calculated indirectly using Engauge Digitizer 4.1 software. Other information included the first author’s name, article date, patient ethnicity, specimen type, test method, cut-off value settings, survival points, follow-up time, quantiles of GDF-15, and other relevant clinicopathological characteristics.

Study quality was judged according to the Quality Assessment of Diagnosis Accuracy Studies criteria (QUADAS), which is based on a 14-item list [25]. The quality of all retrospective cohort studies was assessed using the Newcastle-Ottawa Scale (NOS) checklist, wherein potential bias due to cohort selection, comparability, and outcome ascertainment is judged on a score ranging from 0 to 9 [26]. The included studies were eliminated if they were scored to be of low quality (i.e. a final score of less than 5 for NOS or 8 for Quality Assessment of Diagnostic Accuracy Studies [QUADAS]).

### Statistical analysis

Statistical analyses were conducted using STATA 12.0 software (Stata Corporation, College Station, TX, USA). The primary outcomes (pooled sensitivity, specificity, positive likelihood ratio (PLR), negative likelihood ratio (NLR), diagnostic odds ratio (DOR), and AUC with corresponding 95% CIs) were obtained in the diagnostic meta-analysis; a pooled HR with 95% CI was calculated to measure the association between GDF-15 expression (high vs. low) and the clinical outcomes of patients with DST. A combined HR > 1 implied that GDF-15 had a negative effect on the survival outcome of the patients. Heterogeneity for the size of each effect was calculated using Cochran’s Q and I^2^ statistics, and with statistical significance defined as *P* < 0.05 or I^2^ > 50%. Fixed or random meta-analysis models were selected depending on the degree of study heterogeneity. Influence analysis was undertaken to ascertain the effects of outlier studies on the overall results. Publication bias was examined using Deek’s funnel plot asymmetry test, as well as Egger’s and Begg’s tests, with statistical significance defined as *P* < 0.05.

## Results

### Search results and study quality

**Fig. 1:**
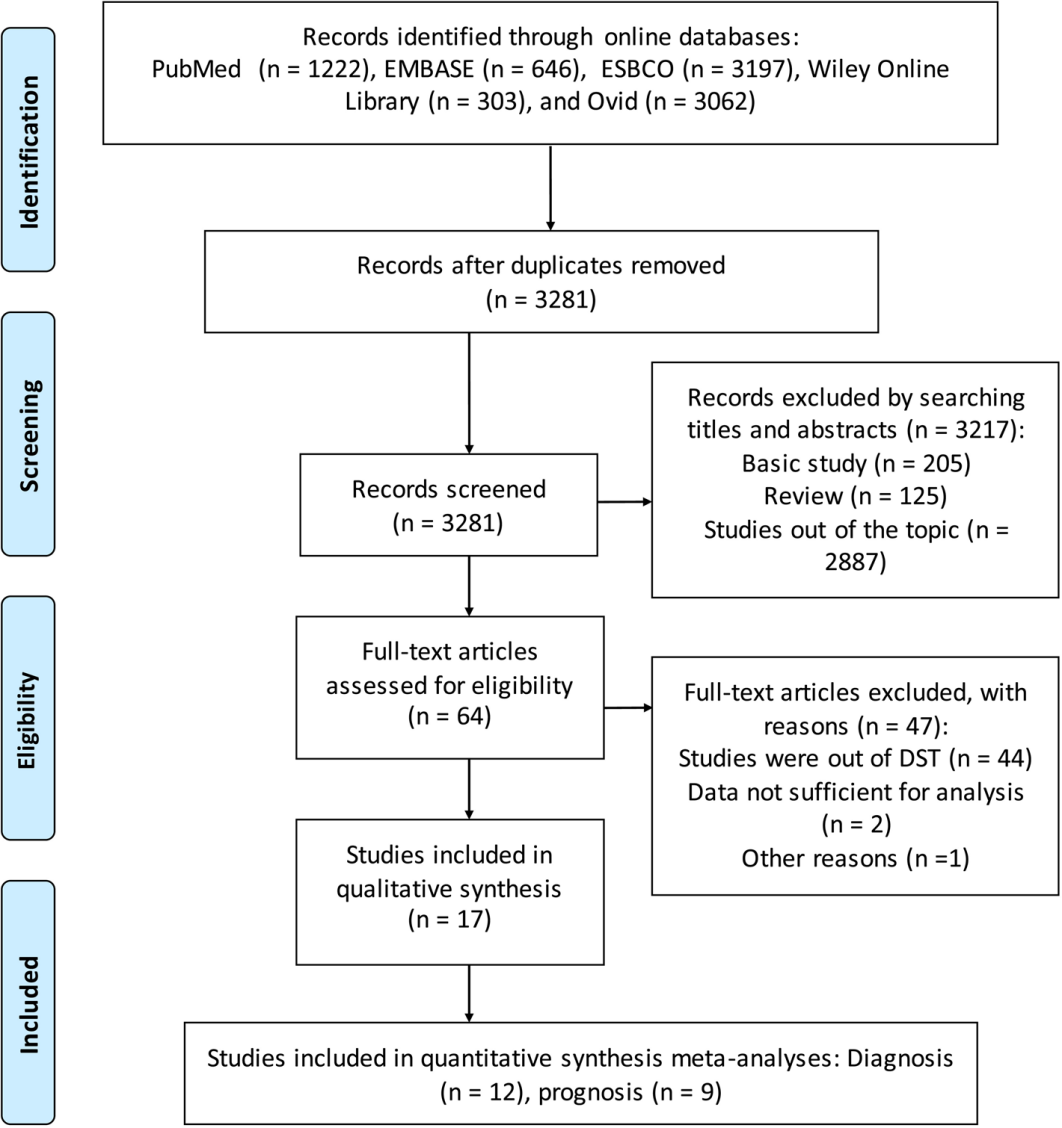
Flow diagram of the study selection and exclusion criteria

Figure 1 schematically displays the selection procedure for eligible articles. According to our search criteria, a total of 3281 studies were eligible after the elimination of duplicates among databases. Among them, 3217 records were excluded due to irrelevant content or non-original data after reading the titles and abstracts. In the subsequent stages of study selection, 64 studies were assessed based on full-text evaluation, and with another 47 excluded. Finally, 17 articles (12 relating to diagnosis, and 9 relating to prognosis) were included in the final meta-analysis.

Study bias judged, as by the 14-item QUADAS list or NOS checklist, revealed that all of the diagnostic and prognostic studies had QUADAS scores of ≥10 or NOS scores of ≥6 (Table 1, Additional file 1 and Additional file 2), indicating that these data were suitable for our final statistical analysis.

**Table 1:** Main features of all included studies used in the diagnostic meta-analysis

Author and reference	Year	Region	Cancer type/cases	Patients TNM (I/II/III/IV)	Control type/number	Test matrix	Method	Cut-off Setting	Sensitivity	Specificity	AUC	QUADAS score
Wang et al. [21]	2014	China	EC/286	48/107/94/37	HC/250	Serum	ELISA	1000 pg/mL	0.381	0.968	0.897	11
Fisher et al. [22]	2015	Australia	EC/40	14/15/8/1	Non-cancer/98	Tissue	MT-PCR	961 pg/mL	0.88	0.64	0.86	10
			EC/30	11/10/8/1	Non-cancer/69	Plasma	ELISA	811 pg/mL	0.83	0.62	0.70	
Blanco-Calvo et al. [15]	2014	Spain	GC/52	9(I-II)/12/31	HC/23	Serum	ELISA	> 325.28 ng/mL	0.7308	0.913	0.8796	10
			GC/52	9(I-II)/12/31	HC/23	Serum	ELISA	> 294.4 ng/mL	0.8889	0.8261	0.884	
Wang et al. [16]	2014	China	PC/807	45/127/337/298	Pancreatitis & benign tumors/165	Serum	ELISA	1000 pg/mL	0.658	0.967	0.739	11
			PC/172	stage I-II: 172	HC/500	Serum	ELISA	1000 pg/mL	0.651	0.95	/	
Koopmann et al. [17]	2006	Australia	PC/50	3/5/39/3	HC/50	Serum	ELISA	1583 pg/mL	0.9	0.84	0.99	10
			PC/50	3/5/39/3	Pancreatitis/50	Serum	ELISA	1583 pg/mL	0.9	0.44	0.81	
Kaur et al. [18]	2013	America	PC/91	5/37/2/38/9(missing)	HC/24	Plasma	ELISA	> 2.3 ng/mL	0.62	0.63	0.85	10
			PC/91	5/37/2/38/9(missing)	Pancreatitis/23	Plasma	ELISA	> 2.3 ng/mL	0.62	0.78	0.74	
			PC/42	Stage I-II: 42	HC/24	Plasma	ELISA	> 2.2 ng/mL	0.81	0.64	0.85	
			PC/49	Stage III-IV: 49	HC/24	Plasma	ELISA	> 1. 6 ng/mL	0.78	0.58	0.94	
			PC/42	Stage I-II: 42	Pancreatitis/23	Plasma	ELISA	> 2.3 ng/mL	0.76	0.78	0.85	
			PC/49	Stage III-IV: 49	Pancreatitis/23	Plasma	ELISA	> 3.5 ng/mL	0.55	0.91	/	
Koopmann et al. [19]	2004	Australia	PC/80	1/3/58/10/8(missing)	Non-cancer/216	Serum	ELISA	1070 pg/mL	0.71	0.78	0.81	10
Xue et al. [12]	2010	China	CRC/144	Stage I-II: 68 Stage III-IV: 76	HC/156	Serum	ELISA	1144 pg/mL	0.778	0.994	0.897	11
Wang et al. [13]	2017	China	CRC/473	51/153/201/68	HC/489	Serum	ELISA	1000 pg/mL	0.438	0.967	0.866	10
Shen et al. [24]	2018	China	Liver cancer/92	18/24/31/19	Benign liver disease: 53 HC: 40	Serum	ELISA	1573.23 ng/L	0.8123	0.8399	/	10
Hogendorf et al. [20]	2018	Poland	PC/42	3/16/9/14	Pancreatitis/21	Serum	ELISA	2.7 ng/mL	0.738	0.7619	/	10
Liu et al. [23]	2015	China	HCC/223	Unclear	Hepatitis/88	Serum	ELISA	2.463 ng/mL	0.631	0.8661	0.7882	11
			HCC/223	Unclear	Non-cancer/290	Serum	ELISA	1.945 ng/mL	0.8679	0.7275	0.8426	

*Abbreviations: MT-PCR* multiplexed tandem PCR, *QUADAS* Quality Assessment for Studies of Diagnostic Accuracy, *EC* esophageal carcinoma, *CRC* colorectal cancer, *HC* healthy control, *GC* gastrointestinal cancer, *PC* pancreatic cancer, *HCC* hepatocellular carcinoma, *AUC* area under the curve, *ELISA* enzyme linked immunosorbent assay

### Study characteristics

The main features of the included studies relating to the diagnostic role of GDF-15 are displayed in Table 1. Twelve diagnostic studies [12, 13, 15–24], comprising 2380 patients and 4630 paired controls, were included in the diagnostic meta-analysis. The study participants involved included Chinese [12, 13, 16, 20, 21, 23, 24], Australian [17, 19, 22], American [18], Polish [20], and Spanish [15] patients, with sample sizes ranging from 42 to 807. The types of DST covered in these studies included colorectal cancer (CRC) [8–13], gastrointestinal cancer (GC) [14, 15], pancreatic cancer (PC) [16–19], esophageal carcinoma (EC) [21, 22], and liver cancer [23, 24], of which the final diagnoses were all confirmed histologically by surgical operation. The types of samples collected included plasma [18, 22], serum [12, 13, 15–17, 19, 21, 23, 24], and tissue [22] samples obtained prior to treatment. Enzyme linked immunosorbent assay (ELISA) was primarily used to test for GDF-15 levels [12, 13, 15–19, 21–24], and only one study used multiplexed tandem PCR (MT-PCR) [22].

We also included 9 cohort studies [8–11, 13–15, 21, 22], with a total sample size of 2200, to assess the association between GDF-15 expression levels (high vs. low) and the clinical outcomes of patients with DST (Table 2). All 9 studies were retrospective, and study populations included Chinese [8, 13, 21], Australian [10, 14, 22], American [11], and Spanish [15] patients. Survival outcomes included OS [8–11, 14, 15, 22], CSS or TSS [11, 13, 21], RFP [21], and PFS [15], with an average follow-up time of 30 months to 9.2 years. In one study [11], HRs calculated based on different quartile points were judged as independent data. However, survival outcomes like RFP and PFS were not combined due to insufficient study numbers.

**Table 2:** Main features of all included studies used in the prognostic meta-analysis

Author & reference	Year	Country	Cancer type	Patient size	Sample type	Method	Expression level	HR	Survival point	*P* value	Follow-up time	extraction	Quartiles of GDF-15	NOS score
Li et al. [8]	2016	China	CRC	138	Serum	ELISA	Increased	1.915	OS	0.045	Unclear	Directly	/	6
Wang et al. [13]	2017	China	CRC	94	Serum	ELISA	Increased	2.917	TSS	0.0005	Average: 43 months	Directly	/	8
								2.607	TSS	0.007				
Wallin et al. [9]	2011	Sweden	CRC	320	Plasma	SP-PLA	Increased	2.11	OS	0.002	Median: 6 years	Directly	/	8
Brown et al. [10]	2003	Australia	CRC	261	Serum	ELISA	Increased	2.2	OS	0.0034	60 months	Directly	/	8
Mehta et al. [11]	2015	America	CRC	618	Plasma	ELISA	Increased	1.88	OS	< 0.0001	Median: 9.2 years	Directly	Quartile2	8
							Increased	1.77	OS	0.0002				
							Increased	1.74	OS	0.0002				
							Increased	1.94	CSS	0.003				
							Increased	1.65	CSS	0.01				
							Increased	1.67	CSS	0.009				
							Increased	2.51	OS	< 0.0001			Quartile3	
							Increased	2.55	OS	0.0002				
							Increased	2.54	OS	0.0002				
							Increased	2.64	CSS	0.003				
							Increased	2.6	CSS	0.01				
							Increased	2.67	CSS	0.009				
							Increased	2.85	OS	< 0.0001			Quartile4	
							Increased	2.63	OS	0.0002				
							Increased	2.63	OS	0.0002				
							Increased	2.73	CSS	0.003				
							Increased	2.34	CSS	0.01				
							Increased	2.4	CSS	0.009				
Fisher et al. [22]	2015	Australia	EC	138	Plasma	ELISA	Increased	2.91	OS	0.076	60 months	Directly	/	9
								3.87	OS	0.048				
Wang et al. [21]	2014	China	ESCC	286	Serum	ELISA	Increased	2.557	TSS	0.002	Average: 30 months	Directly	/	8
								2.625	TSS	0.005				
								1.739	RFS	0.047				
								1.789	RFS	0.050				
Skipworth et al. [14]	2010	Australia	OGC	293	Plasma	ELISA	Increased	1.549	OS	0.036	Over 1500 days	Directly	/	8
Blanco-Calvo et al. [15]	2014	Spain	GC	52	Serum	ELISA	Increased	3.843	OS	0.001	Median:118.9 weeks	Directly	/	8
								3.608	PFS	< 0.001				

*Abbreviations: OGC* oesophago-gastric cancer, *ESCC* esophageal squamous cell carcinoma, *GC* Gastrointestinal cancer, *EC* esophageal carcinoma, *OS* overall survival, *DFS* disease-free survival, *PFS* progression-free survival, *TSS* tumor-specific survival, *CSS* cancer-specific survival, *ELISA* enzyme linked immunosorbent assay, *NOS* Newcastle-Ottawa Scale checklist, *HR* hazard ratio

### Heterogeneity

In the diagnostic meta-analysis, heterogeneity was observed in the overall pooled data, of which the I^2^ value was estimated to be 99.38% (*P* < 0.001). Heterogeneity was also detected among 6 groups in our collected diagnostic data (Table 3), with I^2^ values ranging from 78.4 to 93.7% (*P* < 0.0001). Thus, random effect models were used for these studies. In our pooled data for prognosis, no significant heterogeneity was detected.

**Table 3:** Subgroup analyses of GDF-15 testing for CRC based on different covariates

Analysis	Sensitivity [95% CI]	Specificity [95% CI]	PLR [95% CI]	NLR [95% CI]	DOR [95% CI]	AUC	*ChI*^2^ (P value)/ *I* ^2^
All cancers	0.74 [0.67–0.80]	0.83 [0.75–0.89]	4.45 [2.99–6.60]	0.31 [0.25–0.38]	14.35 [9.13–22.54]	0.85	< 0.001; 99.61%
Cancer type							
PC vs. non-cancer	0.73 [0.66–0.80]	0.76 [0.64–0.85]	3.12 [2.03–4.80]	0.35 [0.27–0.45]	8.92 [5.05–15.77]	0.80	< 0.0001;97.62%
PC vs. HC	0.71 [0.66–0.75]	0.66 [0.62–0.70]	2.21 [1.58–3.11]	0.38 [0.25–0.59]	6.32 [2.75–14.52]	0.73	0.001;78.4%
PC vs. Pancreatitis	0.70 [0.63–0.76]	0.66 [0.57–0.75]	2.81 [1.40–5.64]	0.40 [0.28–0.57]	8.86 [4.81–16.31]	0.81	0.8281;0.0%
PC (stage I-II) vs. non-cancer	0.70 [0.63–0.75]	0.65 [0.61–0.69]	2.04 [1.56–2.68]	0.40 [0.26–0.62]	5.48 [2.58–11.63]	0.76	0.1070;55.2%
PC (stage III-IV) vs. non-cancer	0.66 [0.56–0.76]	0.74 [0.60–0.86]	3.00 [0.82–11.05]	0.47 [0.35–0.62]	6.67 [2.67–16.69]	0.78	0.2984; 7.5%
Esophageal carcinoma	0.47 [0.42–0.53]	0.83 [0.80–0.87]	3.83 [1.46–10.05]	0.34 [0.12–0.93]	13.85 [8.19–23.42]	0.84	0.4586; 0.0%
Sample type							
Plasma	0.69 [0.64–0.73]	0.69 [0.62–0.75]	2.21 [1.79–2.73]	0.44 [0.35–0.54]	6.09 [4.00–9.28]	0.78	0.5158; 0.0%
Serum	0.67 [0.65–0.69]	0.80 [0.78–0.81]	5.25 [3.16–8.71]	0.30 [0.23–0.41]	20.28 [10.12–40.61]	0.87	< 0.0001; 88.9%
Cut-off setting							
≥ 2000 pg/mL (PC vs. non-cancer)	0.66 [0.60–0.71]	0.74 [0.65–0.82]	2.46 [1.70–3.55]	0.46 [0.37–0.57]	6.16 [3.53–10.74]	0.77	0.3172; 15.3%
<2000 pg/mL(PC vs. non-cancer)	0.69 [0.66–0.71]	0.73 [0.70–0.75]	3.30 [1.84–5.93]	0.35 [0.26–0.47]	11.98 [4.42–32.49]	0.85	< 0.0001;90.4%
≥ 2000 pg/mL (all cancers vs. non-cancer)	0.65 [0.60–0.69]	0.80 [0.73–0.85]	2.91 [1.94–4.37]	0.45 [0.39–0.52]	7.34 [4.52–11.91]	0.77	0.2465; 25.0%
<2000 pg/mL (all cancers vs. non-cancer)	0.68 [0.66–0.70]	0.78 [0.76–0.80]	4.36 [2.77–6.85]	0.29 [0.22–0.40]	17.79 [9.00–35.18]	0.87	< 0.0001; 88.1%
Ethnicity							
Caucasian	0.75 [0.71–0.78]	0.71 [0.68–0.75]	2.67 [2.10–3.41]	0.32 [0.25–0.41]	9.94 [6.57–15.04]	0.83	0.0335; 46.4%
Asian	0.65 [0.63–0.67]	0.81 [0.79–0.83]	7.47 [3.21–17.37]	0.36 [0.26–0.52]	22.01 [7.65–63.31]	0.83	< 0.0001;93.7%

*Abbreviations: PLR* positive likelihood ratio, *NLR* negative likelihood ratio, *DOR* diagnostic odds ratio, *AUC* area under the curve, *MT-PCR* multiplexed tandem PCR, *QUADAS* Quality Assessment for Studies of Diagnostic Accuracy, *EC* esophageal carcinoma, *HC* healthy control, *PC* pancreatic cancer, *AUC* area under the curve

### Diagnostic meta-analyses

**Fig. 2:**
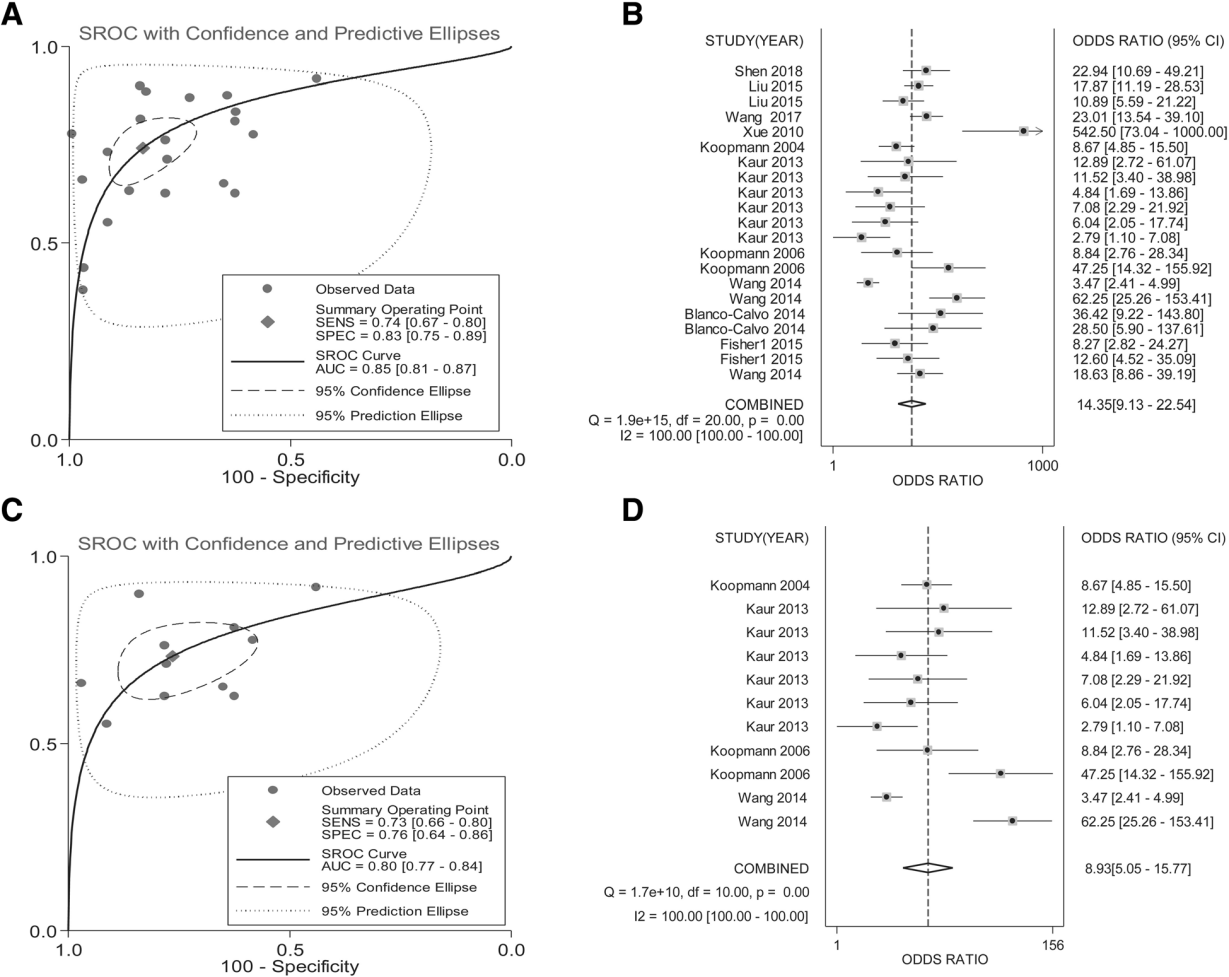
Forest plots of the overall pooled data for GDF-15 (**a**) sensitivity, (**b**) specificity, (**c**) DOR, and (**d**) AUC used to diagnose DST

The overall pooled sensitivity, specificity, diagnostic odds ratio (DOR), and area under the curve (AUC) for GDF-15, used to distinguish DST from non-cancerous tumors, were 0.74 (95% CI: 0.68–0.80), 0.83 (95% CI: 0.75–0.89), 14.07 (95%CI: 9.12–21.71), and 0.84, respectively (Fig. 2 and Table 3), corresponding to a positive likelihood ratio (PLR) of 4.38 (95%CI: 3.00–6.39) and a negative likelihood ratio (NLR) of 0.31 (95%CI: 0.25–0.38). These results suggest that GDF-15 level is a useful alternative biomarker to differentiate patients with DST from those with non-cancerous tumors.

Stratified analyses were performed in the diagnostic meta-analysis based on cancer type, sample type, cut-off setting, and ethnicity. As summarized in Table 3, the pooled AUC of GDF-15 to rule out PC, EC, GC, and liver cancer were estimated to be 0.82, 0.84, 0.90, and 0.85, respectively. Moreover, GDF-15 had an AUC of 0.82 for its ability to distinguish PC from pancreatitis, which was a higher value than the AUC for its ability to distinguish PC from healthy individuals (AUC = 0.73). When meta-analyzed based on sample type, serum-based GDF-15 testing achieved a specificity of 0.80 (95%CI: 0.78–0.81) and an AUC of 0.87, which were superior to plasma-based analysis. We found differences in diagnostic efficacy based on cut-off value: a cut-off setting <2000 pg/mL showed an AUC of 0.85 for PC (PC vs. non-cancerous tumors), and 0.87 for all cancers (all cancers vs. non-cancerous tumors). In the meta-analysis based on ethnicity, GDF-15 testing in Caucasian and Asian patients yielded an AUC of 0.83, whereas the Asian-based test conferred a higher specificity of 0.81 (95% CI: 0.79–0.83). The raw data used for the diagnostic meta-analysis was attached as Additional file 3.

### Prognostic significance

**Fig. 3:**
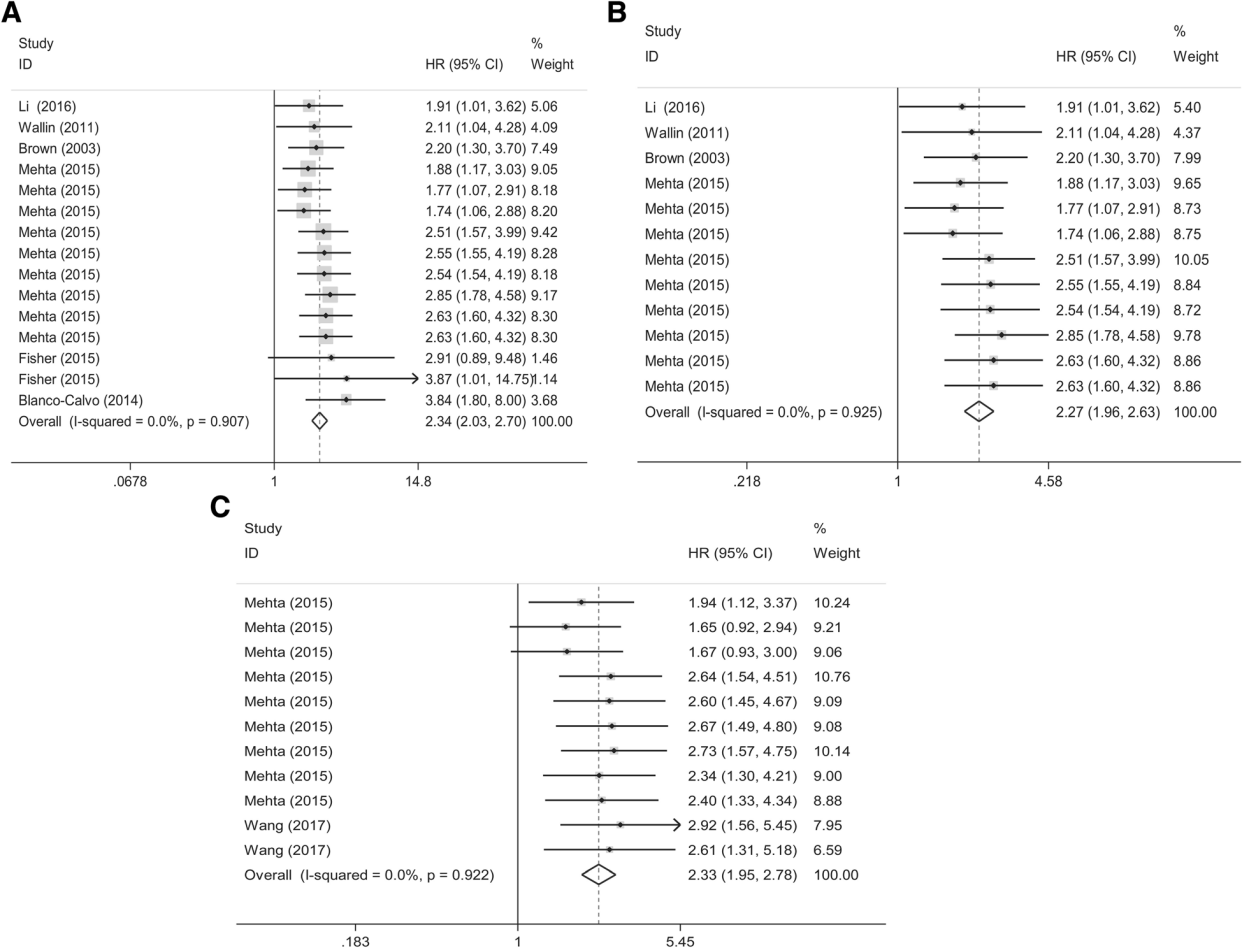
Forest plots of pooled HRs (95% CI) for GDF-15 levels in the prognostic datasets. **a** Pooled HR (95% CI) of OS data for DST; **b** pooled HR (95% CI) of OS data for CRC; **c** pooled HR (95% CI) of CSS/TSS data for CRC

Analysis of a 2200 patient cohort was used to define the association between GDF-15 levels and patient prognosis. Patients with DST who had increased GDF-15 levels had worse overall survival (OS) (HR = 2.34, 95%CI: 2.03–2.70, *P* < 0.001; I^2^ = 0.0%) compared with patients with low GDF-15 levels (Fig. 3a). Moreover, elevated levels of GDF-15 were associated with a significantly shorter OS time in patients with CRC (HR = 2.27, 95%CI: 1.96–2.63, *P* < 0.001; I^2^ = 0.0%) (Fig. 3b). We also included 6 individual data sets for CSS and TSS, and the results showed that GDF-15 levels were correlated with worse CSS in CRC (HR = 2.33, 95% CI: 1.95–2.78, *P* < 0.001; I^2^ = 0.0%)(Fig. 3c). The raw data used for the prognostic meta-analysis was attached as Additional file 4.

### Influence analysis and meta-regression

**Fig. 4:**
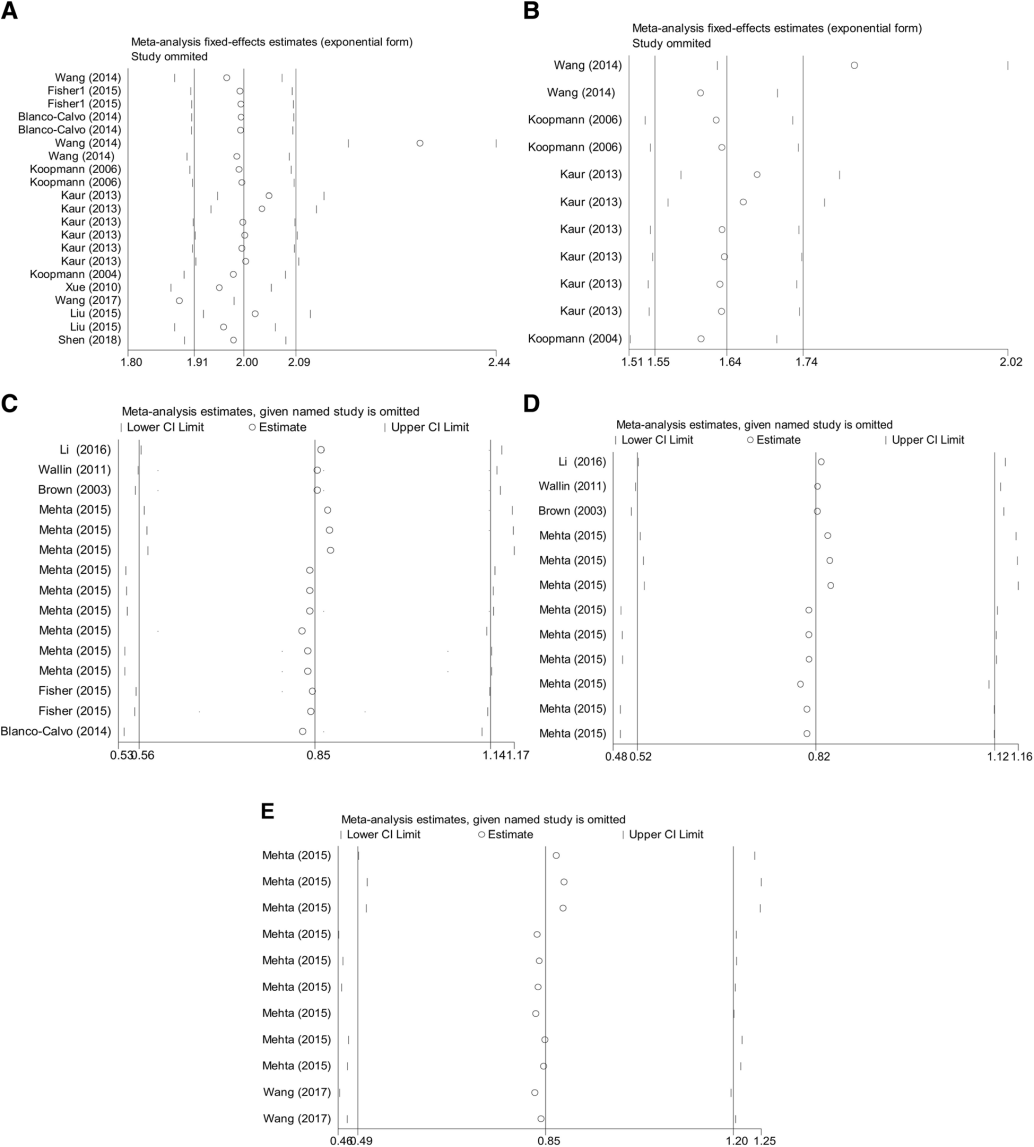
Influence analysis of outlier studies. **a** The overall pooled diagnostic dataset for DST; **b** the combined diagnostic dataset for GDF-15 levels in PC; (**c**) the overall prognostic dataset of OS for DST; **d** the combined prognostic dataset of OS, and CSS (**e**) for GDF-15 in CRC

Influence analysis was conducted for both diagnostic and prognostic meta-analyses using STATA 12.0 software. One individual study [16] was identified as an outlier in the overall pooled diagnostic dataset for DST (Fig. 4a) and PC (Fig. 4b). However, no outlier studies were found at the upper or lower CI limit of the prognostic studies, indicating that the selected studies had relatively high homogeneity (Fig. 4c, d, and e).

Meta-regression was performed to trace the causes of heterogeneity, wherein seven covariates, comprising ethnicity, sample size, control size, cancer type, test matrix, cut-off setting, and QUADAS score, were predefined. As displayed in Additional file 5, the analysis of QUADAS score received the lowest *P*-value (0.0349) among the analyses, suggesting that QUADAS score is the likely source of heterogeneity among diagnostic studies.

### Publication bias

**Fig. 5:**
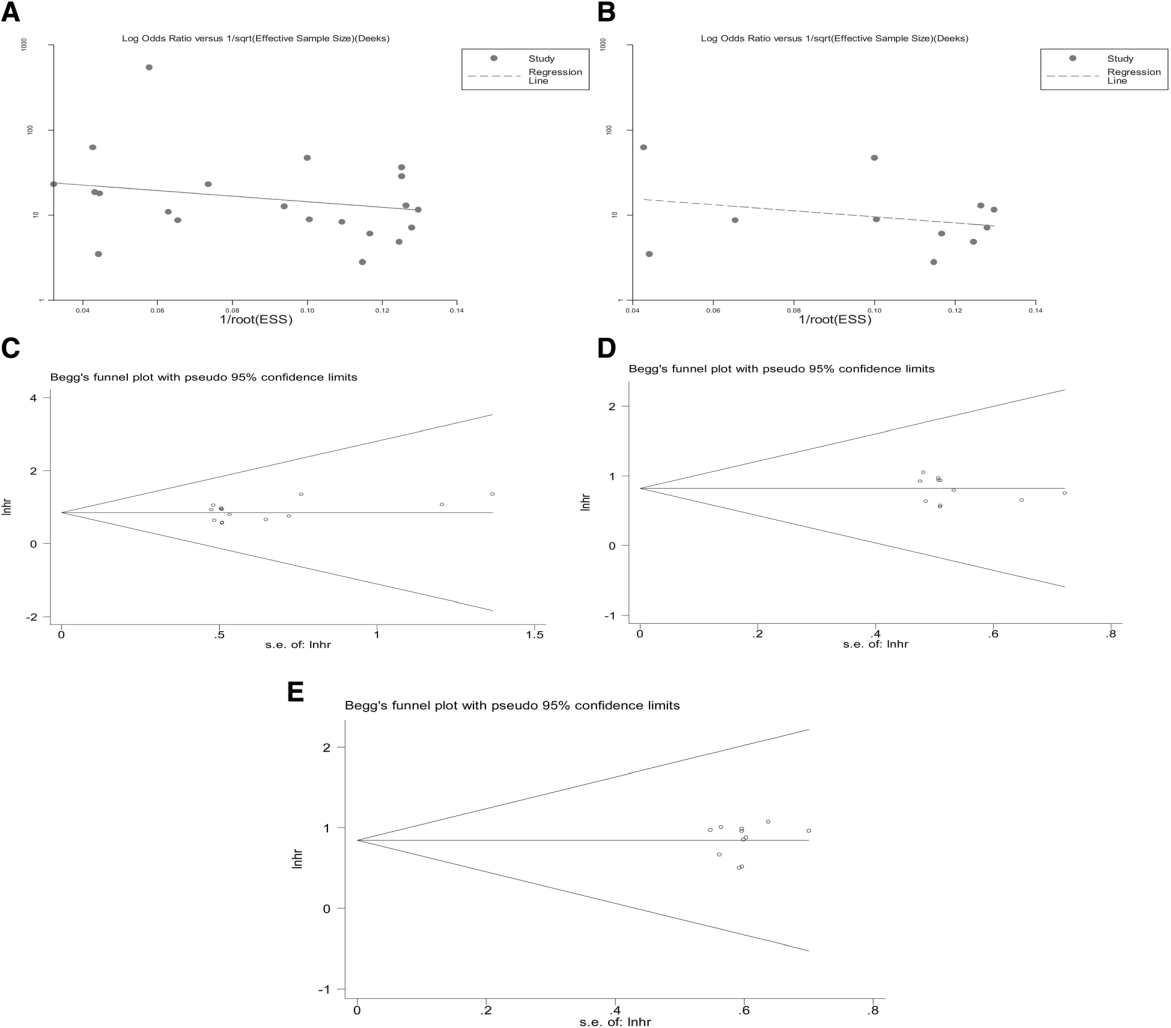
Publication bias judged by Deek’s funnel plot asymmetry test for the overall pooled diagnostic effect of (**a**) DST and (**b**), and Begg’s funnel plot for the overall pooled prognostic effect of (**c**) DST, (**d**) PC, and (**e**) CRC

Publication bias analysis, assessed by Deeks’ funnel plot asymmetry test, demonstrated no clear bias in the overall diagnostic meta-analyses of DST and PC (Fig. 5a and b, *n* = 22 or 12, *P* = 0.375 or 0.479). Additionally, no significant publication bias, as assessed using Egger’s and Begg’s tests, was detected in the meta-analyzed prognostic data (all with *P* > 0.05) (Fig. 5c, d and e).

## Discussion

Expression levels of growth differentiation factor 15 (GDF-15) are increased in most DSTs, including those of the colon [27], stomach [28], pancreas [29], liver [23, 24], and esophagus [21, 22]. Given the important role of GDF-15 in DST, GDF-15 has the potential to be a promising biomarker in DST [6, 8–19, 21–24]. Nevertheless, the utility of GDF-15 as a biomarker has not been confirmed due to a lack of data supported by evidence-based medicine. In the present study, we performed a meta-analysis using recent information obtained regarding GDF-15 as a diagnostic and prognostic biomarker in DSTs.

As expected, GDF-15 was used successfully as a diagnostic biomarker in DST: the pooled sensitivity, specificity, and AUC for the discriminative performance of GDF-15 to rule out DST were 0.74, 0.83, and 0.84, respectively. Although the combined sensitivity was not significantly high, the specificity and AUC were relatively high as well, and illustrated an acceptable diagnostic performance for GDF-15. The diagnostic odds ratio (DOR) is another measure of diagnostic effectiveness, with a value higher than 1.0 representing diagnostic validity [30]. Herein, we obtained a DOR of 14.07, further suggesting that GDF-15 testing can be used to diagnose DST. The pooled PLR of 4.38 also indicated that GDF-15 testing harbored a ratio between the true-positive and false-positive rate.

Several groups have demonstrated that GDF-15 may be used as a biomarker to assist in the detection of PC, EC, GC, and HCC [12, 13, 15–19, 21–24]. In our stratified analysis, 4 groups of carcinomas had been evaluated repeatedly: the pooled AUC of GDF-15 to rule out PC, EC, GC, and HCC were estimated to be 0.82, 0.84, 0.90, and 0.85, respectively, showing that GDF-15 testing achieved a significant level of efficacy in confirming GC. In PC, GDF-15 testing had an AUC of 0.82 for its ability to differentiate PC from pancreatitis, which was higher than its ability to distinguish PC from healthy individuals. These data indicate that GDF-15 may also be a useful indicator for the differential diagnosis of PC and pancreatitis. Additionally, we observed matrix effects for the test performance: serum-based GDF-15 testing yielded a better AUC than that for plasma-based analysis, suggesting that serum samples may be more suitable than plasma samples for GDF-15 testing. We also found differences in diagnostic efficacy based on cut-off value: a cut-off setting of less than 2000 pg/mL exhibited better performance for all cancer types. Lastly, for data stratified by ethnicity, we found an equal diagnostic efficacy of GDF-15 testing between Caucasians and Asians. However, without additional data to support these findings, more investigation is needed.

We found that increased levels of GDF-15 were an independent prognostic marker for DST [8–11, 13–15, 21, 22]. Previously, the topic of whether GDF-15 could serve as prognostic markers for OS, DFS, or RFS in cancer was considered controversial. In our prognostic analysis, 2106 patients with complete follow-up data were included. A clear association between increased GDF-15 levels and shorter OS was observed in patients with DST (HR = 2.34), as well as in colorectal cancer (HR = 2.27). We also included 11 individual studies that measured CSS or TSS in CRC, with results that showed a correlation between GDF-15 expression and poor CSS and TSS (HR = 2.33). These data suggest that GDF-15 could be used as an independent prognostic biomarker in DST. Previous studies have hypothesized that GDF-15 could be used to assist the prediction of cancer recurrence and metastasis in CRC [31, 32]. However, the data obtained for CRC recurrence and metastasis were not sufficient for our study, and were therefore not analyzed.

Study heterogeneity and bias are very common in meta-analysis studies [33]. We observed significant heterogeneity in our diagnostic meta-analyses; thus, we attempted to interpret the cause of this heterogeneity. Firstly, we included studies that included varying patient population. Secondly, patients participating in these studies had different types of cancer and received a wide range of treatments. Moreover, the primary method of GDF-15 expression detection testing (ELISA) used a different cut-off value in each study, particularly that the cut-off points were obviously higher in gastric and liver cancers than other malignancies. Whether the differences in cut-off points were due to cancer type or limited studies still warranted further investigations. Collectively, these factors above may have resulted in non-homogeneous conditions. We therefore conducted sensitivity analysis and meta-regression test. Our sensitivity analysis identified one outlier study, and the degree of heterogeneity was decreased after we excluded all outlier data from the analysis. The univariate meta-regression test showed that only study quality (different QUADAS scores) seemed to be a source of heterogeneity among all other studies.

Limitations of this study include low sample sizes for some cancer types and few available current articles. Secondly, significant heterogeneity was observed in the diagnostic meta-analysis, compromising the overall study accuracy. Lastly, the method used to detect GDF-15 expression consisted primarily of ELISA, which might not be the optimal method to detect GDF-15.

## Conclusions

In summary, we meta-analyzed the diagnostic and prognostic value of GDF-15 in patients with DST. Our analysis provides evidence that elevated GDF-15 levels may be used as a novel diagnostic and prognostic biomarker for DST.

## Additional files

Additional file 1: **Table S1.** Study quality of the diagnostic studies, as assessed by the 14-item QUADAS tool. (DOC 53 kb)

Additional file 2: **Table S2.** Evaluation of bias in the retrospective cohort studies, as assessed using the Newcastle-Ottawa Scale (NOS) checklist. (DOC 36 kb)

Additional file 3: **Table S3.** Raw data used for the diagnostic meta-analysis. The numerical values of TP (true positive), FP (false positive), FN (false negative) and TN (true negative) are available, and were used to construct the 2 × 2 table. (DOC 74 kb)

Additional file 4: **Table S4.** Raw data used for the prognostic meta-analysis. Single HR with 95%CI was extracted or calculated form original studies, and the pooled HR with 95%CI was generated. (DOC 74 kb)

Additional file 5: **Table S5.** Meta-regression test of the overall diagnostic analysis based on different covariates. (DOC 29 kb)

## Abbreviations

AUC: Area under the curve
CRC: Colorectal cancer
CSS: Cancer-specific survival
DFS: Disease free survival
DOR: Diagnostic odds ratio
DST: Digestive system tumors
EC: Esophageal carcinoma
ELISA: Enzyme linked immunosorbent assay
GC: Gastrointestinal cancer
GDF-15: Cytokine growth differentiation factor 15
HC: Healthy control
HCC: Hepatocellular carcinoma
HR: Hazard ratio
MeSH: Medical Subject Headings
MIC-1: Macrophage inhibitory cytokine-1
MT-PCR: Multiplexed tandem PCR
NLR: Negative likelihood ratio
NOS: Newcastle-Ottawa Scale
OS: Overall survival
PC: Pancreatic cancer
PFS: Progression-free survival
PLR: Positive likelihood ratio
QUADAS: Quality assessment for studies of diagnostic accuracy
RFS: Recurrence-free survival
TGF-β1: Transforming growth factor-β
TSS: Tumor-specific survival
