# Supplementary material for: Appraising growth differentiation factor 15 as a promising biomarker in digestive system tumors: a meta-analysis
Source: BMC Cancer. 2019 Feb 26;19:177. doi: 10.1186/s12885-019-5385-y (PMC6390545; doi:10.1186/s12885-019-5385-y)
Supplement: Supplementary file 1 — Table S1. Study quality of the diagnostic studies, as assessed by the 14-item QUADAS tool. (DOC 53 kb) [file 12885_2019_5385_MOESM1_ESM.doc]

Additional file 1: Table S1. Study quality of the diagnostic studies, as assessed by the 14-item QUADAS tool.

| Study | Item 1 | Item 2 | Item 3 | Item 4 | Item 5 | Item 6 | Item 7 | Item 8 | Item 9 | Item 10 | Item 11 | Item 12 | Item 13 | Item 14 |
| --- | --- | --- | --- | --- | --- | --- | --- | --- | --- | --- | --- | --- | --- | --- |
| Wang et al [21] | 1 | 1 | 1 | 1 | 1 | 1 | 1 | 1 | 0 | 1 | 1 | 0 | 0 | 1 |
| Fisher et al [22] | 1 | 1 | 1 | 1 | 1 | 1 | 1 | 0 | 0 | 1 | 1 | 0 | 0 | 1 |
| Blanco-Calvo et al [15] | 1 | 1 | 1 | 1 | 1 | 1 | 1 | 0 | 0 | 1 | 1 | 0 | 0 | 1 |
| Wang et al [16] | 1 | 1 | 1 | 1 | 1 | 1 | 1 | 1 | 0 | 1 | 1 | 0 | 0 | 1 |
| Koopmann et al [17] | 1 | 1 | 1 | 1 | 1 | 1 | 1 | 0 | 0 | 1 | 1 | 0 | 0 | 1 |
| Kaur et al [18] | 1 | 1 | 1 | 1 | 1 | 1 | 1 | 0 | 0 | 1 | 1 | 0 | 0 | 1 |
| Koopmann et al [19] | 1 | 1 | 1 | 1 | 1 | 1 | 1 | 0 | 0 | 1 | 1 | 0 | 0 | 1 |
| Xue et al [12] | 1 | 1 | 1 | 1 | 1 | 1 | 1 | 1 | 0 | 1 | 1 | 0 | 0 | 1 |
| Wang et al [13] | 1 | 1 | 1 | 1 | 1 | 1 | 1 | 0 | 0 | 1 | 1 | 0 | 0 | 1 |
| Shen et al [24] | 1 | 1 | 1 | 1 | 1 | 1 | 1 | 0 | 0 | 1 | 1 | 0 | 0 | 1 |
| Hogendorf et al [20] | 1 | 1 | 1 | 1 | 1 | 1 | 1 | 0 | 0 | 1 | 1 | 0 | 0 | 1 |
| Liu et al [23] | 1 | 1 | 1 | 1 | 1 | 1 | 1 | 1 | 0 | 1 | 1 | 0 | 0 | 1 |
