# Supplementary material for: Appraising growth differentiation factor 15 as a promising biomarker in digestive system tumors: a meta-analysis
Source: BMC Cancer. 2019 Feb 26;19:177. doi: 10.1186/s12885-019-5385-y (PMC6390545; doi:10.1186/s12885-019-5385-y)
Supplement: Supplementary file 2 — Table S2. Evaluation of bias in the retrospective cohort studies, as assessed using the Newcastle-Ottawa Scale (NOS) checklist. (DOC 36 kb) [file 12885_2019_5385_MOESM2_ESM.doc]

Additional file 2: Table S2. Evaluation of bias in the retrospective cohort studies, as assessed using the Newcastle-Ottawa Scale (NOS) checklist.

|  | Cohort selection | | | | Comparability | Outcome ascertainment | | |
| --- | --- | --- | --- | --- | --- | --- | --- | --- |
|  | Representativeness of the exposed cohort | Selection of the non-exposed cohort | Ascertainment of exposure | Demonstration that outcome of interest was not present at start of study | Comparability of cases and controls on the basis of the design or analysis | Assessment of outcome | Was follow-up long enough for outcomes to occur | Adequacy of follow up of cohorts |
| Li et al [8] | 1 | 1 | 1 | 1 | 1 | 1 | 0 | 0 |
| Wang et al [13] | 1 | 1 | 1 | 1 | 2 | 1 | 1 | 0 |
| Wallin et al [9] | 1 | 1 | 1 | 1 | 1 | 1 | 1 | 1 |
| Brown et al [10] | 1 | 1 | 1 | 1 | 1 | 1 | 1 | 1 |
| Mehta et al [11] | 1 | 1 | 1 | 1 | 1 | 1 | 1 | 1 |
| Fisher et al [22] | 1 | 1 | 1 | 1 | 2 | 1 | 1 | 1 |
| Wang et al [21] | 1 | 1 | 1 | 1 | 1 | 1 | 1 | 1 |
| Skipworth et al [14] | 1 | 1 | 1 | 1 | 1 | 1 | 1 | 1 |
| Blanco-Calvo et al [15] | 1 | 1 | 1 | 1 | 1 | 1 | 1 | 1 |
