# Supplementary material for: Appraising growth differentiation factor 15 as a promising biomarker in digestive system tumors: a meta-analysis
Source: BMC Cancer. 2019 Feb 26;19:177. doi: 10.1186/s12885-019-5385-y (PMC6390545; doi:10.1186/s12885-019-5385-y)
Supplement: Supplementary file 3 — Table S3. Raw data used for the diagnostic meta-analysis. The numerical values of TP (true positive), FP (false positive), FN (false negative) and TN (true negative) are available, and were used to construct the 2 × 2 table. (DOC 74 kb) [file 12885_2019_5385_MOESM3_ESM.doc]

Additional file 3: Table S3. Raw data for the diagnostic and meta-analysis.

| Author | Year | SEN | SPE | Patient | Control | cutoff | TP | FP | FN | TN | Cancer | Sample | Control type |
| --- | --- | --- | --- | --- | --- | --- | --- | --- | --- | --- | --- | --- | --- |
| Wang | 2014 | 38.1 | 0.968 | 286 | 250 | 1000pg/ml | 109 | 8 | 177 | 242 | EC | Serum | Normal |
| Fisher1 | 2015 | 0.88 | 0.64 | 40 | 98 | 961 pg/ml | 35 | 35 | 5 | 63 | EC | Tissue | Non-cancer |
| Fisher1 | 2015 | 0.83 | 0.62 | 30 | 69 | 1140 pg ml | 25 | 26 | 5 | 43 | EC | Plasma | Non-cancer |
| Blanco-Calvo | 2014 | 0.7308 | 0.913 | 52 | 23 | >325.28 pg/ml | 38 | 2 | 14 | 21 | GC | Serum | Normal |
| Blanco-Calvo | 2014 | 0.8889 | 0.8261 | 52 | 23 | >294.4 pg/ml | 46 | 4 | 6 | 19 | GC | Serum | Normal |
| Wang | 2014 | 0.658 | 0.967 | 807 | 165 | 1000 pg/mL | 533 | 5 | 274 | 160 | PC | Serum | Benign tumor and pancreatitis |
| Wang | 2014 | 0.651 | 0.95 | 172 | 500 | 1000 pg/mL | 112 | 175 | 60 | 325 | I-IIPC | Serum | Normal |
| Koopmann | 2006 | 0.9 | 0.84 | 50 | 50 | (1,583 pg/mL | 45 | 3 | 5 | 47 | PC | Serum | Normal |
| Koopmann | 2006 | 0.9 | 0.44 | 50 | 50 | (1,583 pg/mL | 45 | 28 | 4 | 22 | PC | Serum | Pancreatitis |
| Kaur | 2013 | 0.62 | 0.63 | 91 | 24 | >2.3ng/ml | 57 | 9 | 34 | 15 | PC | Plasma | Normal |
| Kaur | 2013 | 0.62 | 0.78 | 91 | 23 | >2.3ng/ml | 57 | 5 | 34 | 18 | PC | Plasma | Pancreatitis |
| Kaur | 2013 | 0.81 | 0.64 | 42 | 24 | >2.2ng/ml | 34 | 9 | 8 | 15 | I-II PC | Plasma | Normal |
| Kaur | 2013 | 0.78 | 0.58 | 49 | 24 | >1.6ng/ml | 38 | 10 | 11 | 14 | III-IV PC | Plasma | Normal |
| Kaur | 2013 | 0.76 | 0.78 | 42 | 23 | >2.3ng/ml | 32 | 5 | 10 | 18 | I-II PC | Plasma | Pancreatitis |
| Kaur | 2013 | 0.55 | 0.91 | 49 | 23 | >3.5ng/ml | 27 | 2 | 22 | 21 | III-IV PC | Plasma | Pancreatitis |
| Koopmann | 2004 | 0.71 | 0.78 | 80 | 216 | 1070pg/ml | 57 | 48 | 23 | 168 | PC | Serum | Non-cancer |
| Xue | 2010 | 0.778 | 0.994 | 144 | 156 | 1144 pg/mL | 112 | 1 | 32 | 155 | CRC | Serum | Normal |
| Liu | 2015 | 0.631 | 0.8661 | 223 | 88 | 2.463 ng/mL | 141 | 12 | 82 | 76 | HCC | Serum | Hepatitis |
| Liu | 2015 | 0.8679 | 0.7275 | 223 | 290 | 1.945 ng/mL | 194 | 79 | 29 | 211 | HCC | Serum | Non-cancer |
