# Supplementary material for: Appraising growth differentiation factor 15 as a promising biomarker in digestive system tumors: a meta-analysis
Source: BMC Cancer. 2019 Feb 26;19:177. doi: 10.1186/s12885-019-5385-y (PMC6390545; doi:10.1186/s12885-019-5385-y)
Supplement: Supplementary file 4 — Table S4. Raw data used for the prognostic meta-analysis. Single HR with 95%CI was extracted or calculated form original studies, and the pooled HR with 95%CI was generated. (DOC 74 kb) [file 12885_2019_5385_MOESM4_ESM.doc]

Additional file 4: Table S4. Raw data for the prognostic meta-analysis

| Author | year | HR | LL | UL | P | Cancer | Survival point |
| --- | --- | --- | --- | --- | --- | --- | --- |
| Li | 2016 | 1.915 | 1.014 | 3.617 | 0.045 | CRC | OS |
| Wallin | 2011 | 2.11 | 1.04 | 4.28 |  | CRC | OS |
| Brown | 2003 | 2.2 | 1.3 | 3.7 | 0.0034 | CRC | OS |
| Mehta | 2015 | 1.88 | 1.17 | 3.03 |  | CRC | OS |
| Mehta | 2015 | 1.77 | 1.07 | 2.91 |  | CRC | OS |
| Mehta | 2015 | 1.74 | 1.06 | 2.88 |  | CRC | OS |
| Mehta | 2015 | 1.94 | 1.12 | 3.37 |  | CRC | CSS |
| Mehta | 2015 | 1.65 | 0.92 | 2.94 |  | CRC | CSS |
| Mehta | 2015 | 1.67 | 0.93 | 3 |  | CRC | CSS |
| Mehta | 2015 | 2.51 | 1.57 | 3.99 |  | CRC | OS |
| Mehta | 2015 | 2.55 | 1.55 | 4.19 |  | CRC | OS |
| Mehta | 2015 | 2.54 | 1.54 | 4.19 |  | CRC | OS |
| Mehta | 2015 | 2.64 | 1.54 | 4.51 |  | CRC | CSS |
| Mehta | 2015 | 2.6 | 1.45 | 4.67 |  | CRC | CSS |
| Mehta | 2015 | 2.67 | 1.49 | 4.8 |  | CRC | CSS |
| Mehta | 2015 | 2.85 | 1.78 | 4.58 |  | CRC | OS |
| Mehta | 2015 | 2.63 | 1.6 | 4.32 |  | CRC | OS |
| Mehta | 2015 | 2.63 | 1.6 | 4.32 |  | CRC | OS |
| Mehta | 2015 | 2.73 | 1.57 | 4.75 |  | CRC | CSS |
| Mehta | 2015 | 2.34 | 1.3 | 4.21 |  | CRC | CSS |
| Mehta | 2015 | 2.4 | 1.33 | 4.34 |  | CRC | CSS |
| Fisher | 2015 | 2.91 | 0.89 | 9.48 |  | EC | OS |
| Fisher | 2015 | 3.87 | 1.01 | 14.75 |  | EC | OS |
| Blanco-Calvo | 2014 | 3.843 | 1.799 | 8 | 0.001 | EC | OS |
| Wang | 2017 | 2.917 | 1.561 | 5.452 |  | CRC | CSS |
| Wang | 2017 | 2.607 | 1.312 | 5.181 |  | CRC | CSS |
