# Supplementary material for: Appraising growth differentiation factor 15 as a promising biomarker in digestive system tumors: a meta-analysis
Source: BMC Cancer. 2019 Feb 26;19:177. doi: 10.1186/s12885-019-5385-y (PMC6390545; doi:10.1186/s12885-019-5385-y)
Supplement: Supplementary file 5 — Table S5. Meta-regression test of the overall diagnostic analysis based on different covariates. (DOC 29 kb) [file 12885_2019_5385_MOESM5_ESM.doc]

Additional file 5: Table S5. Meta-regression test of the overall diagnostic analysis based on different covariates.

| Covariates | Std. Err. | P value | PDOR [95% CI] |
| --- | --- | --- | --- |
| Cancer types | 0.1964 | 0.7610 | 1.06 [0.70-1.61] |
| Test matrix | 0.2292 | 0.0846 | 0.66 [0.40-1.07] |
| Ethnicity | 0.5753 | 0.6790 | 0.78 [0.23-2.69] |
| Patient size | 0.5496 | 0.8408 | 0.89 [0.28-2.87] |
| Control size | 0.6101 | 0.8925 | 1.09 [0.29-4.06] |
| Cut-off setting | 0.4334 | 0.1947 | 0.56 [0.22-1.39] |
| QUADAS score | 0.6486 | 0.0349 | 4.5 [1.13-17.93] |
